# Predicting human protein subcellular localization by heterogeneous and comprehensive approaches

**DOI:** 10.1371/journal.pone.0178832

**Published:** 2017-06-28

**Authors:** Chi-Hua Tung, Chi-Wei Chen, Han-Hao Sun, Yen-Wei Chu

**Affiliations:** 1 Department of Bioinformatics, Chung-Hua University, Hsinchu, Taiwan; 2 Institute of Genomics and Bioinformatics, National Chung Hsing University 250, Taichung 402, Taiwan; 3 Biotechnology Center, Agricultural Biotechnology Center, Institute of Molecular Biology, Graduate Institute of Biotechnology, National Chung Hsing University 250, Taichung 402, Taiwan; Harbin Institute of Technology Shenzhen Graduate School, CHINA

## Abstract

Drug development and investigation of protein function both require an understanding of protein subcellular localization. We developed a system, REALoc, that can predict the subcellular localization of singleplex and multiplex proteins in humans. This system, based on comprehensive strategy, consists of two heterogeneous systematic frameworks that integrate one-to-one and many-to-many machine learning methods and use sequence-based features, including amino acid composition, surface accessibility, weighted sign aa index, and sequence similarity profile, as well as gene ontology function-based features. REALoc can be used to predict localization to six subcellular compartments (cell membrane, cytoplasm, endoplasmic reticulum/Golgi, mitochondrion, nucleus, and extracellular). REALoc yielded a 75.3% absolute true success rate during five-fold cross-validation and a 57.1% absolute true success rate in an independent database test, which was >10% higher than six other prediction systems. Lastly, we analyzed the effects of Vote and GANN models on singleplex and multiplex localization prediction efficacy. REALoc is freely available at http://predictor.nchu.edu.tw/REALoc.

## Introduction

Current efforts in genomics science routinely make use of various fast and accurate sequencing platforms. These technologies have led to greater understanding of protein function and the regulation of biological networks, and the rapidly accumulated sequence information has allowed exploration of complex physiological mechanisms and numerous diseases. Protein localization in cells is often closely correlated with its function. According targeting signals may occur anywhere in the protein sequence, proteins will be sorted to their destination. For example, signal peptide is a short N-terminal amino acid sequence that guide the distribution of the protein to the membrane of the endoplasmic reticulum (ER) and enter the secretory pathway [1,2]. Therefore, we endeavored to use the available sequence information to provide quick subcellular localization prediction. Two transduction pathways exist: Co-translational translocation involves signal-recognition particles and the endoplasmic reticulum delivery system, whereas in post-translational translocation, translation is completed in the cytoplasm, but the protein retains information specifying delivery to different organelles [3]. Nakai *et al*. (1991) first proposed the prediction of subcellular localization via signal peptides. However, due to the very recent growth of protein databases and development of different machine learning methods, TargetP and SignalP 4.0 are currently the only commonly known prediction systems [4,5], yet they can only predict localization to three subcellular compartments (chloroplast, mitochondrion, and extracellular).

Methods for predicting subcellular localization can be classified into the following three types: 1) Homology-based prediction compares the localization of known proteins with unknown proteins. If a certain degree of similarity is found in the sequence, then it can be inferred that the unknown protein’s subcellular localization may be the same as the known protein [6]. The Basic Local Alignment Search Tool (BLAST) sequence alignment method [7] has been widely applied in several prediction systems, including WegoLoc, iLoc-Hum, and Euk-mPloc 2.0 [8–10]. However, when the similarity between the unknown protein and the database is low, this method has poor predictive ability. Hence, it is often integrated with other methods. 2) Functional domain-based prediction relies on known structures or functional data, such as protein functional domains and motifs, as well as information in the gene ontology (GO) database [6,8,11–14]. There are many learning models of research methods are used to establish the relevance of GO terms and subcellular localization. [10,12,15–19] It has been shown that GO terms can be used to advance the performance of subcellular localization prediction. These functional data regarded as domain knowledge are highly accurate and reliable, but this approach requires manual verification of each annotation and cannot be applied to the whole new protein; therefore it is usually combined with the homology-based approach. The optimized combined approach can greatly increase the predictive accuracy. 3) Sequence-based prediction relies on information about the primary amino acid sequence of proteins used for information technology operations or discovery of hidden information, commonly including amino acid composition, pseudo amino acid composition, and n-grams [19–22]. Prediction results from this approach are typically less informative than those for homology- and functional domain-based methods, but in predicting subcellular localization of unknown proteins, it is still a feasible approach. There are also systems based on n-grams for processing the amino acid sequence to analyze the subcellular location of the target protein [21]. Most of the existing methods described above only predict singleplex proteins, those that localize to a single subcellular location, and they do not consider multiplex proteins, those that localize to two or more different compartments within the cell. Identifying multiple locations of a multiplex protein has high value to understanding biological functions, and there is much room for continued development in this area [23].

Currently, the development of subcellular location prediction tools faces two major problems: the difference among the data quantity of locations is too large and the poor predictive ability for multiplex proteins [24]. Therefore, a highly accurate system was developed for predicting human protein subcellular localization that we called REALoc (Reliable and Effective methods to Assist predicting human protein subcellular Localization), which consists of two systematic frameworks. The first level uses 32 support vector machine (SVM) models in a one-to-one relationship system to completely cover all negative learning information by comprehensive strategy; the second level uses a many-to-many relationship system comprised of two learning mechanisms, a genetic algorithm optimized neural network (GANN) and majority voting (Vote), which represent the strong and weak data correlation, respectively. REALoc can predict localization to the cell membrane, cytoplasm, endoplasmic reticulum/Golgi, mitochondrion, nucleus, and extracellular. With regards to the learning sequences, this study adopted not only the commonly used amino acid composition (AAC) and surface accessibility (SA), but also two new learning features, weighted sign amino acid index (aa index) and sequence similarity profile. Weighted sign aa index allows more emphasis to be placed on subcellular localization-related amino acid features in the learning process, while sequence similarity profile provides identifiable learning information for similar sequences in proteins with different subcellular localization. In regards to structural learning features, we used the regular maximum relevance minimum redundancy (regular-mRMR) method to select the 35 best localization- related sets of features for GO.

An independent testing dataset was used to verify the predictive power of REALoc, and the prediction results were compared to six current predictors, CELLO [25], locTree2 [26] iLoc-hum [14], Hum-mPLoc 2.0 [27], GOASVM [6] and mGOF-loc [22]. REALoc also uses the absolute true success rate (ATSR) [9,14,24] to represent the protein prediction score, which avoids under- and over-prediction. Using the systematic ATSR evaluation method we objectively evaluated the reliability of the human subcellular localization prediction system. After five-fold cross-validation, the ATSR with the training data and independent testing data used in this study were 75.3 and 56.5%, respectively. Whether a protein was located in a single or multiple subcellular compartments, REALoc showed higher overall performance compared with other prediction systems, with a >10% improvement in the ATSR in independent testing. In terms of the analysis of the REALoc system, we also removed the GO information to evaluate the predictive power for novel proteins, and the prediction efficacies of the Vote and GANN learning mechanisms for singleplex and multiplex protein data were also analyzed.

## Materials and methods

### Data processing

#### Training dataset 5939p

The dataset was obtained from the UniProtKB/Swiss-Prot protein database October 2011 version [28]. Proteins with a comment on subcellular location were extracted and those with uncertain terms such as “by similarity,” potential,” and “probable” were removed. Sequences <80 amino acids in length were also removed. CD-HIT was used [29] to eliminate redundant sequences by setting the threshold to 0.7, whereas other parameters were set as the default. Note that if the threshold of similarity is set too high, the learning model will easily lead to overfitting. On the other hand, lower threshold value will be resulting in poor learning effect. Data were divided into six types, cell membrane, cytoplasm, endoplasmic reticulum/Golgi, mitochondrion, nucleus and extracellular (Table 1). There were total of 5939 different proteins in the training dataset, and multiplex proteins accounted for 15% of the entire dataset (S1 Fig). In other parts of subcellular location, such as centrosome, cytoskeleton, endosome, lysosome, microsome, peroxisome and synapse, the number of the training set is too fewer to obtain accurate prediction [16].

**Table 1:** Number of proteins in the different subcellular locations in the training and testing datasets.

Subcellular location	Training dataset	Testing dataset
Cell membrane	1453	221
Cytoplasm	1542	197
ER/Golgi	562	136
Mitochondrion	462	133
Nucleus	2064	156
Extracellular	795	82
Total	6878^a^ (5939^b^)	925^a^ (868^b^)

^a^Total number of proteins in all locations

^b^Total number of different proteins

#### Testing dataset 868pt

The UniProtKB/Swiss-Prot protein database April 2013 version was acquired [28], and the proteins in the 5939p training dataset were removed. CD-HIT was then used again to remove repetition. The details for the subcellular locations of the resulting dataset are shown in Table 1. There were 868 different proteins, among which 44% were multiplex proteins (S1 Fig).

### Sequence-based features

#### AAC and SA

Certain protein regions closely correlate with subcellular localization. Protein sequences can be divided into full-length, the first 30 N-terminal amino acids, the middle third of the protein, and the last 50 C-terminal amino acids. The AAC and SA were calculated for each of these regions for each protein. The AAC equation (Eq 1) was as follows:

AAC(ai)=(totalnumberofaminoacidai)N,ai∈20aminoacids (1)

where *a*_*i*_ represents the 20 amino acids and N represents the full length of the given region. Basic SA information was obtained via NetSurfP [30], individually considering the relative and accessible surface area and amino acid exposure for the four regions.

#### Weighted sign aa index

There are 96 different amino acid characteristics, such as hydrophobicity, polarity and transfer free energy [31,32], that have been used to increase the accuracy of predicting subcellular localization. We included an additional four indices with the same amino acid characteristics but from different studies to obtain 100 amino acid indices (S1 Table) that were used to calculate an index value for each protein. After obtaining the index, we used the AAC for the four regions to assess the AAindex for the different sequence regions and calculated the weighted sign aa index with the equations:

$$H=\{h_{1}h_{2}\cdots h_{20}\},\;C_{i}\in \{c_{1}{,c}_{2},c_{3},\cdots ,c_{100}\}$$ (2)

$${score}_{i}=\sum_{j=1}^{20}AAC(a_{j})\times h_{j}$$ (3)

$$C_{i}=log({|score}_{i}|)$$ (4)

$$\begin{array}{l} weighted\;sign\;aa\;index=\{\left({sign}_{1},C_{1}),({sign}_{2},C_{2}),\ldots ,({sign}_{100},C_{100}\right)\},\\ where\;{sign}_{i}=\left\{ \begin{array}{c} 1,\;\mathrm{if}\;({score}_{i}<0)\\ 0,\;\mathrm{if}\;({score}_{i}\geq 0) \end{array}\right. \end{array}$$ (5)

In Eq 2, *H* indicates the set of 20 different amino acid index values corresponding to each amino acid, and *C*_*i*_ indicates one of 100 amino acid indices we collected. Then, AAC was used to derive *score*_*i*_ (Eq 3), and these scores were then normalized by obtaining the logarithms (*C*_*i*_, Eq 4). After calculating *sign*_*i*_, it was combined with *C*_*i*_ to obtain the weighted sign aa index (Eq 5).

#### Pseudo amino acid composition (PseAAC)

Chou *et al*. (2001) reported that, in addition to examining the composition of the standard 20 amino acids, investigating PseAAC provides order information of the amino acids for more complete retention of protein sequences. Also, state-of-the-art in the studies on pseudo K-tuple nucleotide composition have noted that the concept can apply to the field of DNA and RNA sequence analysis. The study in iEnhancer-PsedeKNC provide excellent discussions of the applications of regulatory DNA elements identification [33]. The research of iDHS-EL [34] and iRSpot-EL [35] addressed important claims regarding prediction of DNase I hypersensitive sites and recombination hotspot in a genome by using pseudo nucleotide composition and other sequence-based features into classifier. Besides, iMiRNA-PseDPC has pointed out that the new feature called "pseudo distance-pair composition" have a primary role in identifying the human pre-miRNAs efficiently for large-scale genome analysis [36].

PseAAC provides order information of the elements for more complete retention of sequences. Chou *et al*. (2015) integrated four PseAAC modes for protein sequences in the web server Pse-in-One [37]. For heterogeneous and comprehensive learning, Realoc do not consider the two modes of PC- and SC-PseAAC-general because they were extracted from AAindex which is the same source as the feature weighted sign aa index. On the other hand, the mode PC-PseAAC contains one more physicochemical index than SC-PseAAC, called side chain. Therefore, the mode PC-PseAAC in the Pse-in-One was adopted as PseAAC encoding by Realoc. We applied the approach of Chou *et al*., with a 0.05 weight factor ω and λ of 8 [20,38].

#### Sequence similarity profile (SSP)

To address the issue of proteins having different subcellular locations despite high similarity in sequences, we considered secondary sequence-based features for certain proteins. As Chen *et al*. (2016) noted in their review of protein remote homology detection, sequence similarity is a major concern in a wide variety of computational methods [39]. In this research, BLAST was used to infer sequence similarity between query protein and training dataset. It compares each protein with 5939p and obtain three sequence-based features described above from the most similar sequence, which we refer to as the SSP. If a similar protein could not be found, then the sequence-based features of the query protein would be the SSP.

### Function-based features

The GO database does not provide any sequence data. To simplify data handling, a GO sequence database integrating the 2013_0318 version of GO with the UniProtKB/Swiss-Prot protein database was established. A protein often has several GO numbers. In the feature-selection method, acquiring appropriate GO numbers can effectively assist in the learning of the prediction model. We revised the standard mRMR feature-selection algorithm [40] (S1 Algorithm) to obtain the top 50 GO numbers in order of relevance to subcellular localization, which were then used as the feature set in prediction using the SVM model. The results showed that optimum prediction accuracy was obtained with the first 35 GO numbers. Thus, the first 35 GO numbers were adopted for function-based features (S2 Fig).

### System implementation

**Fig 1:**
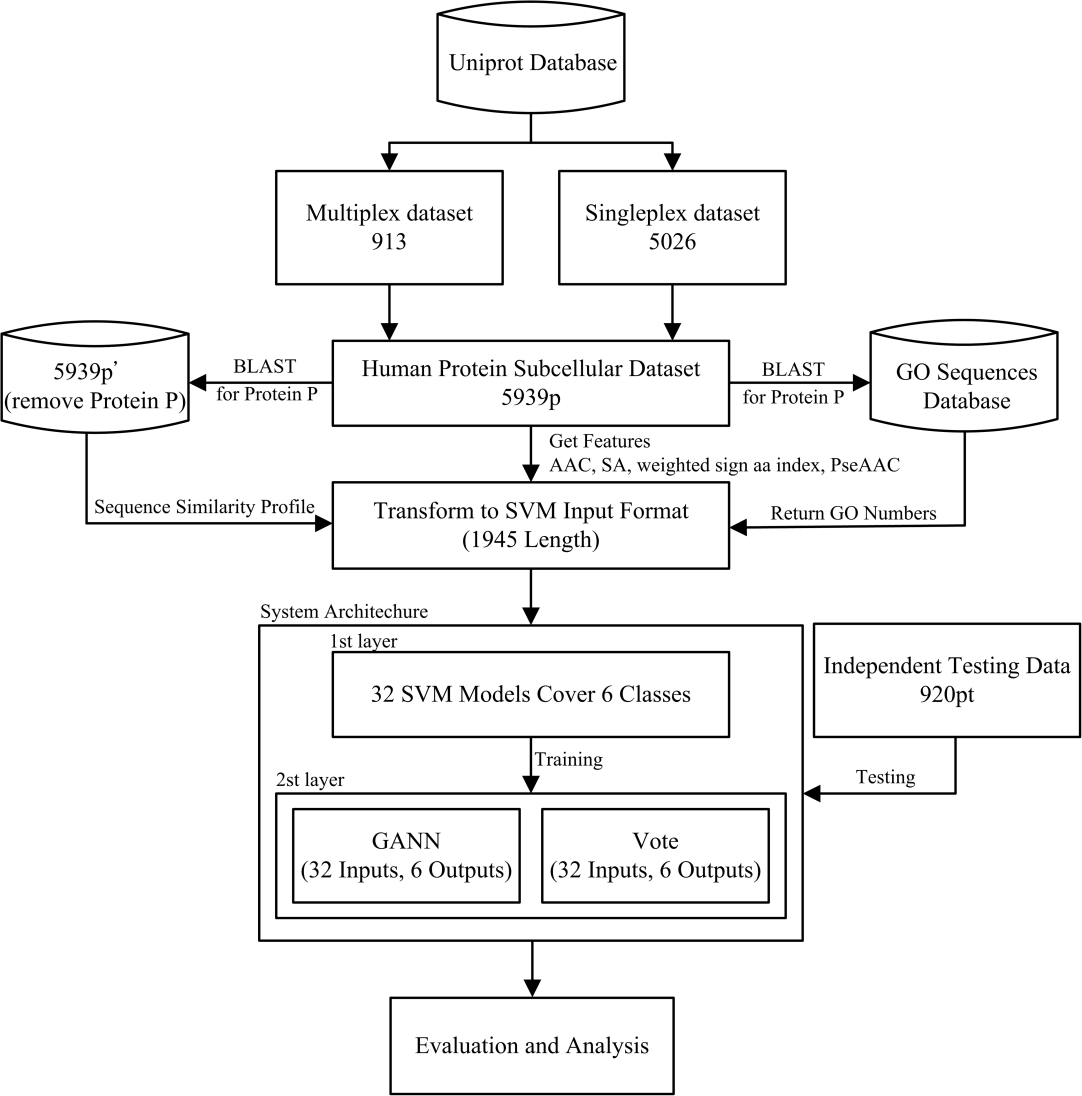
The flowchart shows the implementation of REALoc.

A schematic overview of the system is shown in Fig 1. The proteins in the 5939p training dataset were obtained from UniProtKB, then sequence-based features were collected and calculated and NetSurfP was used to obtain SA prediction results. The query sequence was BLASTed against 5939p to obtain an SSP, and AAC, PseAAC, and weighted sign aa index were calculated using defined equations. In the function-based features collection, the primary sequence of proteins was used to find similar proteins in the GO sequence database, and these GO numbers were added to the feature set for that protein.

After the features were obtained and values calculated, the values were converted into SVM input format. The fixed input length was 1945. They were then provided to each SVM for the first layer of learning. The input for the second learning layer was the output of the first layer SVMs, using GANN or Vote mechanisms to integrate the input results and for validation and analysis. We also prepared the independent testing dataset 868pt to compare the prediction accuracy of REALoc for singleplex and multiplex proteins with those of other prediction systems.

### Two-layer framework

**Fig 2:**
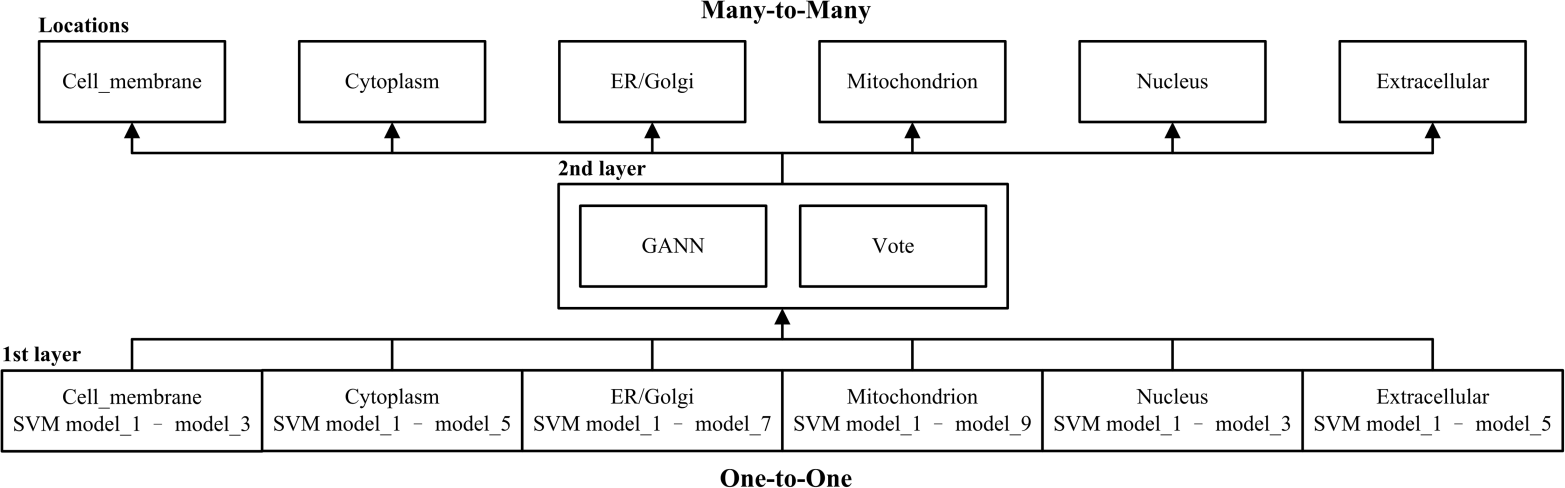
Two-layer architecture of REALoc.

REALoc can be divided into two kinds of relationship layers, one-to-one and many-to-many (Fig 2). The first layer consists of 32 SVMs based on training data for six subcellular locations. Each location has three to nine SVM models that were obtained based on the best positive to negative ratio (S2 Table). The second layer contains GANN and Vote models. GANN used the first layer SVM results as training data to predict the six subcellular locations, whereas Vote used SVMs from different locations for majority voting. The first layer intentionally used an odd number of models for easy operation.

#### First layer: SVM models

SVMs are widely used in subcellular localization studies. Some studies have used hierarchical structure SVMs [26,41] or the balanced multi-model [42]. However, when there are large differences in the quantity of source data, it can lead to bias in the prediction. Therefore, this study designed various subcellular localization classifications so that they are based on multiple SVM models, and each sub-predictor underwent optimal adjustment for the data ratio (S3–S8 Figs). Moreover, full negative data were adopted for learning to obtain optimum learning outcomes without losing information. The first layer of REALoc was constructed with LIBSVM [43].

#### Second layer: GANN and vote

Previous studies have combined neural networks and genetic algorithms to deal with classification problems that could prevent neural networks from falling into local optimum [44]. REALoc adopted a GANN model using the SVM results from the first layer to predict the possible subcellular locations and establish a many-to-many prediction core. The parameters settings of the GANN model were population = 500, mutation = 0.3, crossover = 0.7, maxW = 25, minW = -25, maxGen = 1000, and error = 0.05. Vote used multiple SVM models for evaluation. The second layer of Vote predicts six subcellular localizations based on particular subsets of SVM prediction models. When more models showed agreement than opposition for a particular subcellular location, the query protein was predicted to appear in that location.

### Cross-validation and performance evaluation

Because REALoc was composed of two layers of prediction models, the learning models in the two layers were evaluated and verified using a five-fold cross-validation mechanism (S9 Fig). The absolute true success rate, ATSR, was assessed as following equation (Eq 6). ATSR is a prediction evaluation measure that requires successful prediction for a protein in all subcellular locations where it could be found. ATSR is stricter than the other evaluation indicators, because the predictive score of the test protein can be counted as 1 if and only if all of its subcellular positions are accurately predicted without any under-prediction or over-prediction [9,14,24].

$$\begin{array}{l} Absolut\;true\;success\;rate=\frac{\sum_{i=1}^{N}\Delta (i)}{N},\\ where\;\Delta (i)=\left\{ \begin{array}{c} 1,\;ith\;protein\;are\;correctly\;predicted\\ 0,\;otherwise \end{array}\right. \end{array}$$ (6)

## Results and discussion

### The performances of REALoc and other predictors in 5939p

In order to realize and validate our training model is mature enough to achieve the standard level of the current prediction tools, REALoc used the training dataset 5939p and five-fold cross-validation to verify its learning and predictive power for unknown proteins. The prediction results from REALoc were compared to those of other prediction tools for 5939p (Table 2).

**Table 2:** Performance of REALoc with five-fold cross-validation and other predictors for the 5939p dataset.

Subcellular location	LocTree2	CELLO	Hum-mPLoc 2.0	iLoc-hum	REALoc_ Vote	REALoc_ GANN
Cell membrane	57.5	58.9	61.8	69.7	79.6	**80.6**
Cytoplasm	30.0	25.2	45.3	**66.6**	63.5	65.6
ER/Golgi	38.1	2.1	53.9	32.4	74.4	**81.1**
Mitochondrion	51.8	57.1	66.7	**84.3**	76.0	75.3
Nucleus	57.3	58.8	71.1	71.6	72.7	**74.8**
Extracellular	78.7	62.1	74.8	80.1	76.5	**80.9**
Overall	60.0	54.3	67.2	72.7	72.8	**75.3**

All results are the absolute true success rate given as %, and the bold text indicates the highest value in each row.

REALoc obtained results with the Vote and GANN learning mechanisms in the second layer. The ATSR of REALoc_Vote was 72.8%, and the best prediction result was 75.3% by the REALoc_GANN learning model. Compared to the GANN learning model, Vote is more intuitive, but it does not consider the correlation between the sub-models in the first layer. Therefore, in terms of learning, the GANN model was better for 5939p.

The overall ATSRs of LocTree2 and CELLO were 60.0 and 54.3%, respectively. LocTree2 is a newer system developed in 2012 and therefore achieves better performance. In terms of the prediction results for human proteins, Hum-mPLoc 2.0 and iLoc-hum showed better accuracy, with ATSRs of 67.2and 72.7%, respectively. Among the six subcellular locations, iLoc-hum was most accurate at predicting localization to the cytoplasm and mitochondrion, whereas REALoc_GANN obtained superior results for the remaining four subcellular locations.

### The performances of REALoc and other predictors for 868pt

Proteins that localize to multiple subcellular localizations accounted for ~44% of the independent test dataset 868pt. Thus, this dataset could be used to evaluate the ability of each system to identify multiplex proteins (Table 3). REALoc_Vote had the highest accuracy of 57.1%, followed by REALoc_GANN at 52.5%. For individual subcellular localizations, iLoc-hum was the best at predicting localization to mitochondrion, with an accuracy of 64.7%, GOASVM had the highest accuracy of 94.9% for predicting nuclear localization, and REALoc_Vote performed best for the remaining four subcellular location predictions, with accuracy ranging from 53 to 58% for cell membrane, cytoplasm and ER/Golgi localization and reaching the maximum accuracy of 80% for prediction of extracellular localization.

**Table 3:** Testing dataset 868pt predicted by REALoc and other predictors.

Subcellular location	LocTree2	CELLO	Hum-mPLoc 2.0	iLoc-hum	GOASVM	mGOF-loc	REALoc_ Vote	REALoc_ GANN
Cell membrane	23.3	30.8	29.4	43.9	6.8	28.6	**57.9**	47.5
Cytoplasm	8.0	8.1	22.3	35.5	4.1	7.6	**57.4**	50.8
ER/Golgi	18.9	0.7	28.9	22.1	3.7	0.0	**53.7**	52.9
Mitochondrion	38.8	30.1	45.9	**64.7**	11.3	25.2	53.4	54.1
Nucleus	58.5	64.7	63.3	57.7	**94.9**	89.9	58.3	55.8
Extracellular	50.0	40.0	52.0	40.0	76.0	40.0	**80.0**	**80.0**
Overall	28.4	27.4	36.9	44.0	24.2	30.4	**57.1**	52.5

All results are the absolute true success rate given as % (the number of proteins correctly predicted/the number of total accepted proteins), and the bold text indicates the highest value in each row.

From the analyses of 5939p and 868pt, iLoc-hum showed good predictive power for mitochondrial localization. In addition, in the 868pt dataset, the nuclear-localized proteins showed the highest ratio of singleplex to multiplex proteins, accounting for 89% (139/156). Therefore, for subcellular localization to the nucleus, GOASVM, the singleplex-based system, had more accurate predictions than other, multiplex-based systems.

### The predictive ability for multiplex proteins

In regards to the prediction results of multiplex proteins with the training dataset 5939p (Table 4), REALoc_GANN had optimal prediction accuracy of 75.9%, whereas Hum-mPLoc 2.0, iLoc-hum and mGOF-loc showed 30.5%, 44.3% and 6.4% accuracy, respectively, far lower than REALoc. As for the multiplex protein validation with the independent testing dataset 868pt, REALoc_Vote performed the best, with a 50.1% ATSR. In the test excluding GO information, REALoc_Vote had the best performance, with an ATSR of 30.1%. The other prediction systems used data for 14 or 37 different subcellular locations as training information. Because more locations result in more complicated data, the prediction accuracy of these two systems for the six locations in this study was lower. In contrast, although REALoc can only predict these six locations, because the training dataset had more protein sequences and the one-to-one learning mechanism we proposed addressed issues of imbalanced data, REALoc showed excellent predictive performance for multiplex proteins.

**Table 4:** Performance of REALoc and other approaches for predicting multiplex proteins.

Dataset	Hum-mPLoc 2.0	iLoc-hum	mGOF-loc	REALoc_ Vote	REALoc_ GANN
5939p	30.5 (278/912)	44.3 (404/913)	6.4 (55/854)	74.2 (677/913)	**75.9** (693/913)
868pt	20.0 (80/400)	17.3 (70/405)	2.7 (10/375)	**50.1** (203/405)	36.3 (147/405)
868pt (without GO)	n/a*	n/a*	n/a*	**30.1** (122/405)	22.7 (92/405)

* Not available

All results are the absolute true success rate given as % (the number of proteins correctly predicted/the number of total accepted proteins), and the bold text indicates the highest value in each row.

### The comparison of REALoc_GANN and REALoc_Vote

To compare the second layer prediction models of REALoc_GANN and REALoc_Vote, 5939p and 868pt prediction performance results were analyzed in terms of singleplex and multiplex proteins (Table 5). The five-fold cross-validation for 5939p showed that the GANN model had better performances for singleplex and multiplex proteins. This is because the quantity of singleplex protein is 69.2% more than that of multiplex proteins in the 5939p dataset, causing an increase in overall prediction. However, the percentage of quantity of the multiplex protein in 868pt was 29% higher than that of the multiplex protein in 5939p, accounting for 44% of the independent testing dataset. With this dataset, REALoc_Vote had a prediction performance 14% better than REALoc_GANN for multiplex proteins (50.1 and 36.3% ATSR, respectively). However, for singleplex proteins, the predictive power of REALoc_GANN was still better (3.4% higher than REALoc_Vote).

**Table 5:** Performance comparison of REALoc_GANN and REALoc_Vote.

Detaset	REALoc_Vote	REALoc_GANN
Training dataset (5939p) Singleplex	72.5 (3645/5026)	**75.3** (3782/5026)
Training dataset (5939p) Multiplex	74.2 (677/913)	**75.9** (693/913)
Training dataset (5939p) Overall	72.8 (4322/5939)	**75.3** (4475/5939)
Testing dataset (868pt) Singleplex	63.3 (293/463)	**66.7** (309/463)
Testing dataset (868pt) Multiplex	**50.1** (203/405)	36.3 (147/405)
Testing dataset (868pt) Overall	**57.1** (496/868)	52.5 (456/868)

All results are representative as absolute true success rate (%), given by (the number of proteins correctly predicted / the number of total accepted proteins).

GANN learning incorporates structural parameters with genetic algorithms to predict possible subcellular locations, which can provide good predictive power with the relatively simple learning requirements for singleplex prediction. On the other hand, neural networks are based on insufficient information to complete the more complex task of predicting multiplex proteins. In addition, the predictive power of majority voting strategies come from the efficacies of each SVM model in the first layer. More accurate first layer SVM models equate to more accurate voting. Therefore, REALoc_Vote is more suitable for multiplex prediction.

## Conclusions

Here we describe a prediction system, REALoc, that can be used to predict human protein localization to six major subcellular locations: the cell membrane, cytoplasm, endoplasmic reticulum/Golgi, mitochondrion, nucleus, and extracellular. REALoc combines various sequence-based analysis features, such as AAC, PseAAC, SA, and the weighted sign aa index and SSP we developed, as well as GO function-based features, to provide highly accurate reliable prediction results. In addition, in consideration of the recent appreciation of multiplex protein prediction as well as the issues of large differences in the amounts of source data and poor prediction power of current systems for multiplex proteins, two learning mechanisms with different characteristics were used (one-to-one SVMs and many-to-many GANN) to establish a two-layer prediction system. On the other hand, the best positive to negative ratio was found to avoid great effect upon data amount, and then using multiple one-to-one models to consider all negative data without losing any information.

The ATSRs for the training dataset 5939p and testing dataset 868pt were 75.3 and 57.1%, respectively, and the prediction results for 5939p and 868pt were 2.6 and 13.1% higher than those of other prediction systems, respectively. This study further determined that the predictive power of REALoc for multiplex proteins was twice that of current existing prediction tools. This may be due to a decrease in classifications caused an increase in learning accuracy and the fact that there was enough data to provide REALoc with well learning and system stability.

REALoc can only predict six subcellular locations, less than some other prediction systems. In the future we will focus on developing prediction methods applicable for smaller sets of data to increase the coverage of subcellular localizations in REALoc. In addition, REALoc, like other commonly used prediction methods, uses BLAST to compare GO information when analyzing unknown proteins. However, because BLAST is based on local alignment, if complete information on protein sequence is to be considered, future studies should adopt global alignment or comparison methods to increase the accuracy in using GO features.

REALoc provides two prediction models, Vote and GANN, which had different efficacies for different protein characteristics. We suggest using GANN for singleplex proteins and Vote for multiplex proteins, and this approach has been incorporated into the default settings in the freely accessible public REALoc online server at http://predictor.nchu.edu.tw/REALoc.

## Supporting information

S1 Fig The percentage of two kinds of proteins in training and testing dataset. The percentage of singleplex and multiplex proteins in the dataset.
(PDF)

S2 Fig The performance of REALoc with GO feature selection by regular-mRMR at six kinds of locations. To determine feature numbers used in REALoc.
(PDF)

S3 Fig Negative and positive ratio test in Cell membrane by SVMs. Select the best ratio used in SVMs.
(PDF)

S4 Fig Negative and positive ratio test in Cytoplasm by SVMs. Select the best ratio used in SVMs.
(PDF)

S5 Fig Negative and positive ratio test in ER/Golgi by SVMs. Select the best ratio used in SVMs.
(PDF)

S6 Fig Negative and positive ratio test in Mitochondrion by SVMs. Select the best ratio used in SVMs.
(PDF)

S7 Fig Negative and positive ratio test in Nucleus by SVMs. Select the best ratio used in SVMs.
(PDF)

S8 Fig Negative and positive ratio test in extracellular by SVMs. Select the best ratio used in SVMs.
(PDF)

S9 Fig Cross-validation. The flowchart of two layer 5-fold cross-validation in REALoc.
(PDF)

S1 Table AAindex list. List of amino acid indices were used in REALoc.
(PDF)

S2 Table Model number and Best P/N ratio. The number of SVM models and the ratio of negative and positive data in the first layer.
(PDF)

S1 Algorithm Regular-mRMR. The procedure of regular-mRMR in REALoc.
(PDF)
