# Supplementary figures and images for: Predicting human protein subcellular localization by heterogeneous and comprehensive approaches
Source: PLoS One. 2017 Jun 28;12(6):e0178832. doi: 10.1371/journal.pone.0178832 (PMC5489166; doi:10.1371/journal.pone.0178832)

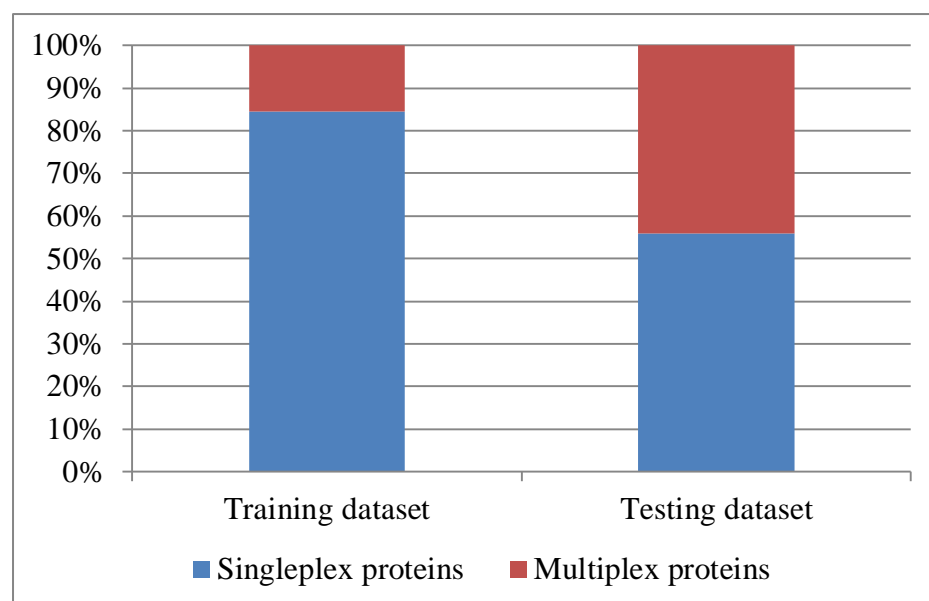

**Supplementary figure 1** The percentage of two kinds of proteins in training and testing dataset

Supplement: S1 Fig — The percentage of singleplex and multiplex proteins in the dataset. (PDF) [file pone.0178832.s001.pdf]

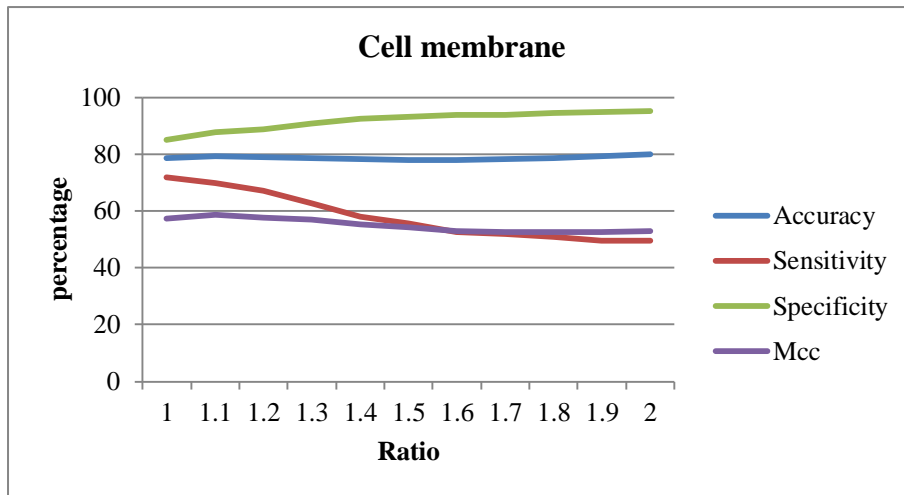

**Supplementary figure 3** Negative and positive ratio test in Cell membrane by SVMs

Supplement: S3 Fig — Select the best ratio used in SVMs. (PDF) [file pone.0178832.s003.pdf]

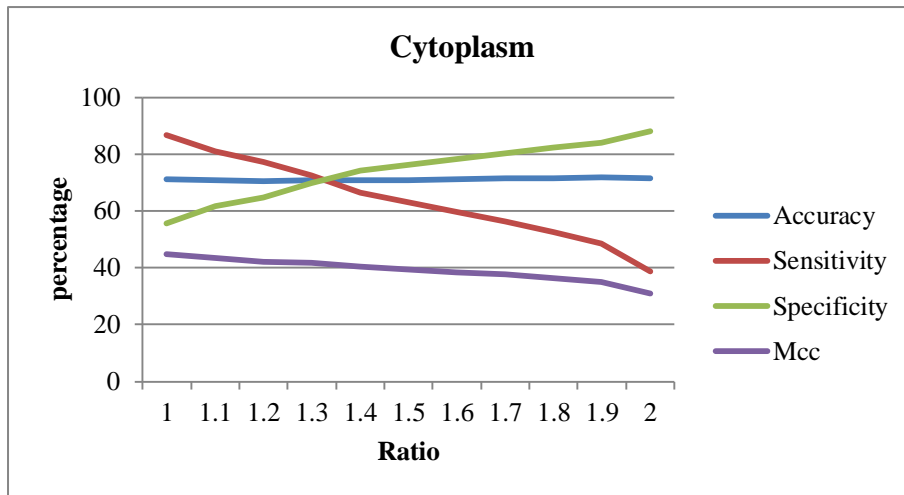

**Supplementary figure 4** Negative and positive ratio test in Cytoplasm by SVMs

Supplement: S4 Fig — Select the best ratio used in SVMs. (PDF) [file pone.0178832.s004.pdf]

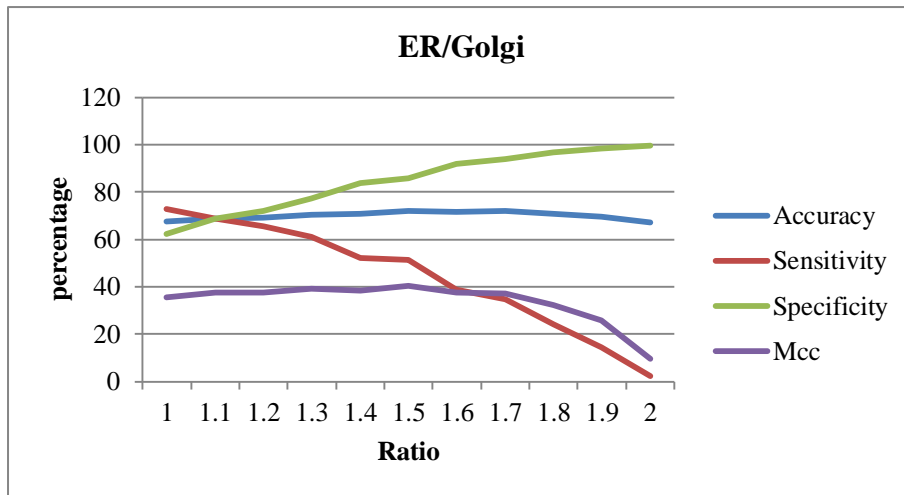

**Supplementary figure 5** Negative and positive ratio test in ER/Golgi by SVMs

Supplement: S5 Fig — Select the best ratio used in SVMs. (PDF) [file pone.0178832.s005.pdf]

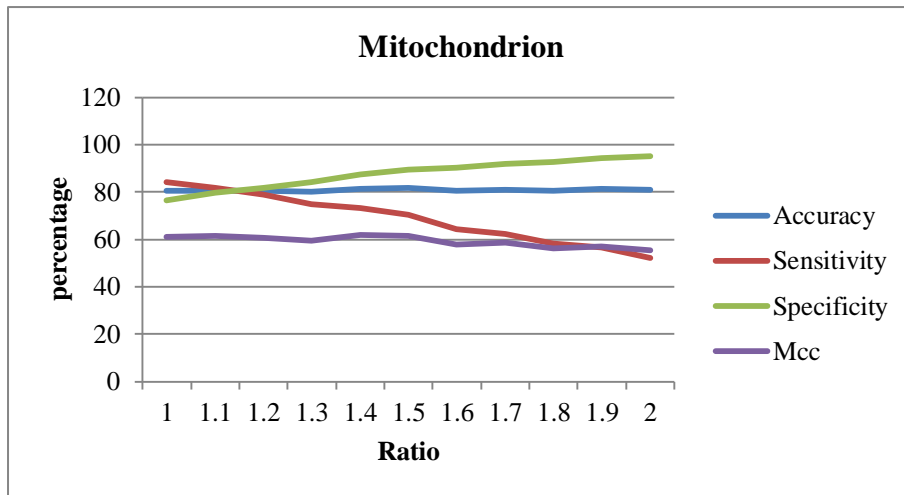

**Supplementary figure 6** Negative and positive ratio test in Mitochondrion by SVMs

Supplement: S6 Fig — Select the best ratio used in SVMs. (PDF) [file pone.0178832.s006.pdf]

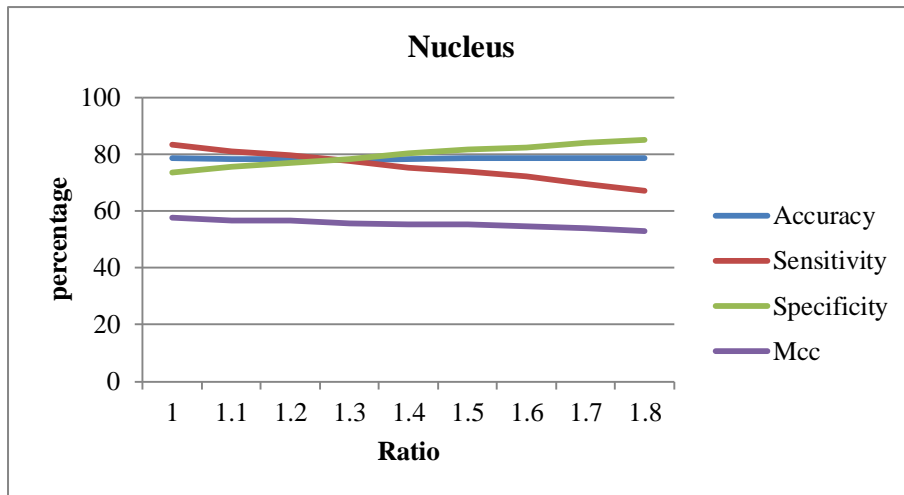

**Supplementary figure 7** Negative and positive ratio test in Nucleus by SVMs

Supplement: S7 Fig — Select the best ratio used in SVMs. (PDF) [file pone.0178832.s007.pdf]

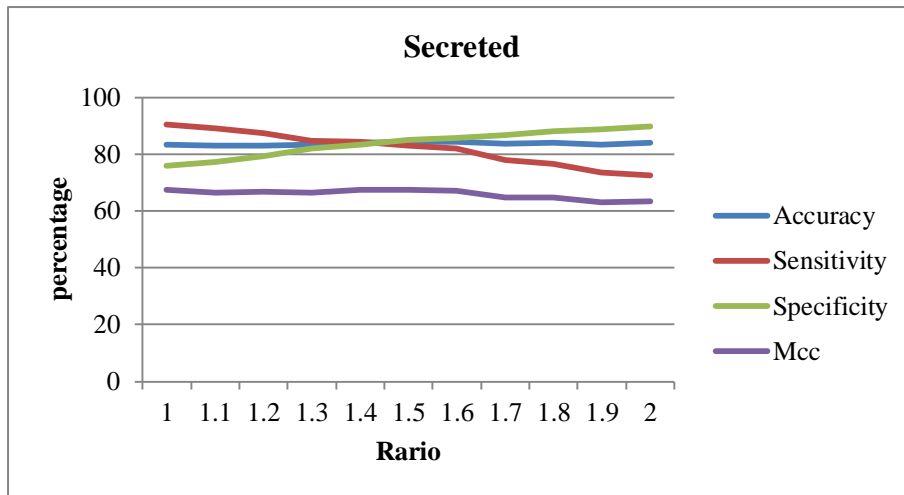

**Supplementary figure 8** Negative and positive ratio test in extracellular by SVMs

Supplement: S8 Fig — Select the best ratio used in SVMs. (PDF) [file pone.0178832.s008.pdf]
