# Supplementary material for: Predicting human protein subcellular localization by heterogeneous and comprehensive approaches
Source: PLoS One. 2017 Jun 28;12(6):e0178832. doi: 10.1371/journal.pone.0178832 (PMC5489166; doi:10.1371/journal.pone.0178832)
Supplement: S2 Fig — To determine feature numbers used in REALoc. (PDF) [file pone.0178832.s002.pdf]

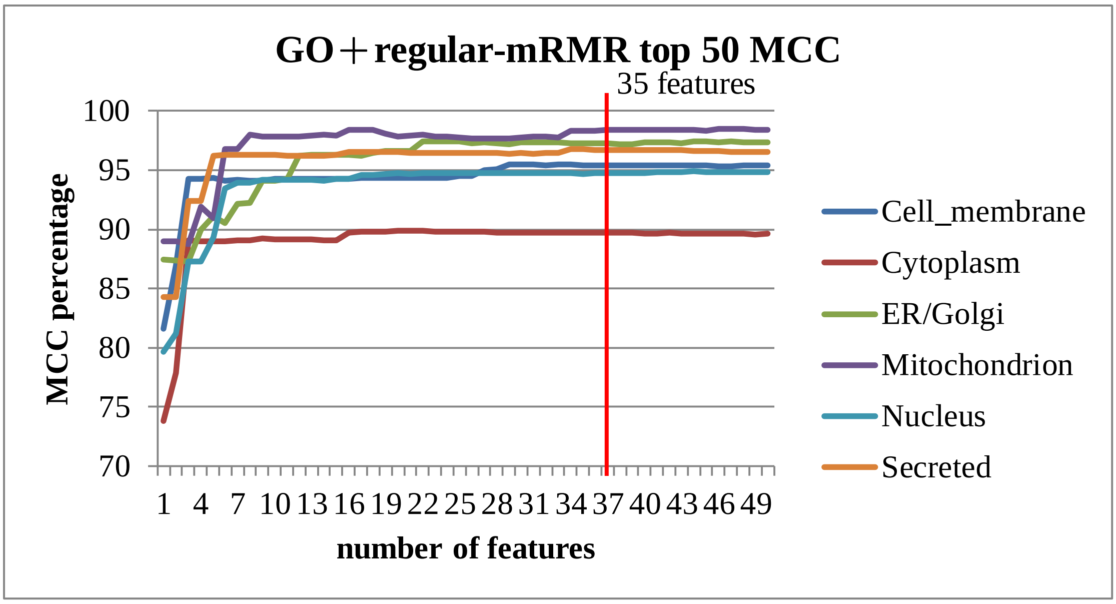

Supplementary figure 2 The performance of REALoc with GO feature selection by regular-mRMR at six kinds of locations
