# Supplementary material for: Predicting human protein subcellular localization by heterogeneous and comprehensive approaches
Source: PLoS One. 2017 Jun 28;12(6):e0178832. doi: 10.1371/journal.pone.0178832 (PMC5489166; doi:10.1371/journal.pone.0178832)
Supplement: S9 Fig — The flowchart of two layer 5-fold cross-validation in REALoc. (PDF) [file pone.0178832.s009.pdf]

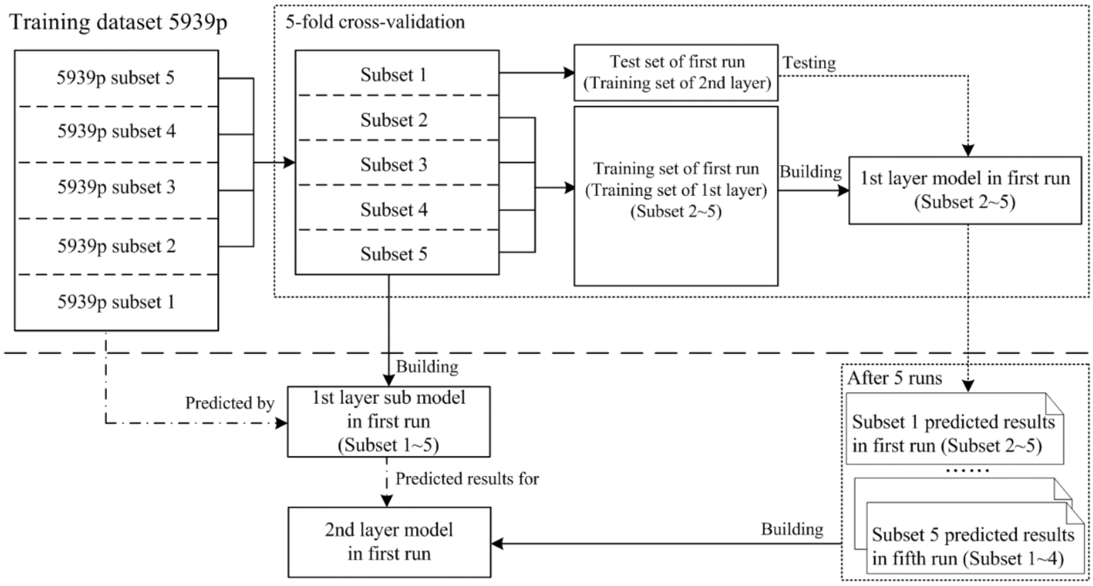

Supplementary figure 9 Building the training dataset for 5-fold cross-validation. The 5939p was first divided into five parts for 5-fold cross-validation to assess the accuracy of the two levels. For examples, the first execution used one-fifth of the data (5939p subset 1) to test the accuracy from the first layer to the second layer. The remaining four-fifths of the data (5939p subsets 2-5) were used as the training data for the first prediction model and 5-fold cross-validation was used to create the training data for the second prediction model. The framework accuracy for the first one-fifth of the data (5939p subset 1) was obtained after the first and second layer predictions. This reasoning was used to obtain the cross-validation results for the two-layer model.
