# Supplementary material for: Predicting human protein subcellular localization by heterogeneous and comprehensive approaches
Source: PLoS One. 2017 Jun 28;12(6):e0178832. doi: 10.1371/journal.pone.0178832 (PMC5489166; doi:10.1371/journal.pone.0178832)
Supplement: S1 Table — List of amino acid indices were used in REALoc. (PDF) [file pone.0178832.s010.pdf]

Supplementary table 1 List of amino acid indices were used in REALoc

Accession number	Data description
**ANDN920101**	alpha-CH chemical shifts
**ARGP820101**	Hydrophobicity index
**BIGC670101**	Residue volume
**BROC820101**	Retention coefficient in TFA
**BULH740101**	Transfer free energy to surface
**BULH740102**	Apparent partial specific volume
**BUNA790101**	alpha-NH chemical shifts
**BUNA790102**	alpha-CH chemical shifts
**BUNA790103**	Spin-spin coupling constants 3JHalpha-NH
**BURA740101**	Normalized frequency of alpha-helix
**BURA740102**	Normalized frequency of extended structure
**CHAM810101**	Steric parameter
**CHOP780201**	Normalized frequency of alpha-helix
**DAWD720101**	Size
**DAYM780201**	Relative mutability
**FASG760101**	Molecular weight
**GEIM800104**	Alpha-helix indices for alpha/beta-proteins
**GEIM800105**	Beta-strand indices
**GEIM800107**	Beta-strand indices for alpha/beta-proteins
**GOLD730101**	Hydrophobicity factor
**GOLD730102**	Residue volume
**GRAR740101**	Composition
**GRAR740102**	Polarity
**GUYH850101**	Partition energy
**HUTJ700101**	Heat capacity
**HUTJ700102**	Absolute entropy
**HUTJ700103**	Entropy of formation
**ISOY800101**	Normalized relative frequency of alpha-helix
**ISOY800102**	Normalized relative frequency of extended structure
**ISOY800103**	Normalized relative frequency of bend
**ISOY800104**	Normalized relative frequency of bend R
**ISOY800107**	Normalized relative frequency of double bend
**ISOY800108**	Normalized relative frequency of coil
**JANJ780102**	Percentage of buried residues
**JANJ780103**	Percentage of exposed residues
**JANJ790101**	Ratio of buried and accessible molar fractions (36)

**JANJ790102**	Transfer free energy
**JOND750101**	Hydrophobicity
**JOND920101**	Relative frequency of occurrence
**JOND920102**	Relative mutability
**JUKT750101**	Amino acid distribution
**JUNJ780101**	Sequence frequency
**KANM800101**	Average relative probability of helix
**KANM800102**	Average relative probability of beta-sheet
**KANM800103**	Average relative probability of inner helix
**KARP850101**	Flexibility parameter for no rigid neighbors
**KARP850102**	Flexibility parameter for one rigid neighbor
**KARP850103**	Flexibility parameter for two rigid neighbors
**KHAG800101**	The Kerr-constant increments
**KLEP840101**	Net charge
**KRIW710101**	Side chain interaction parameter
**KRIW790101**	Side chain interaction parameter
**KRIW790102**	Fraction of site occupied by water
**KRIW790103**	Side chain volume
**KYTJ820101**	Hydropathy index
**LEVM760101**	Hydrophobic parameter
**LEVM760103**	Side chain angle theta(AAR)
**LEVM760105**	Radius of gyration of side chain
**LEVM760107**	van der Waals parameter epsilon
**LEWP710101**	Frequency of occurrence in beta-bends
**LIFS790102**	Conformational preference for parallel beta-strands
**MAXF760101**	Normalized frequency of alpha-helix
**MAXF760102**	Normalized frequency of extended structure
**MAXF760103**	Normalized frequency of zeta R
**MAXF760104**	Normalized frequency of left-handed alpha-helix
**MAXF760105**	Normalized frequency of zeta L
**MEEJ810101**	Retention coefficient in NaClO4
**MEEJ810102**	Retention coefficient in NaH2PO4
**MEIH800102**	Average reduced distance for side chain
**MEIH800103**	Average side chain orientation angle
**MIYS850101**	Effective partition energy
**NAGK730101**	Normalized frequency of alpha-helix
**NAGK730102**	Normalized frequency of bata-structure
**NAGK730103**	Normalized frequency of coil

**NAKH900101**	AA composition of total proteins
**NAKH900102**	SD of AA composition of total proteins
**NAKH900103**	AA composition of mt-proteins
**NAKH900108**	Normalized composition from fungi and plant
**NAKH920101**	AA composition of CYT of single-spanning proteins
**NAKH920103**	AA composition of EXT of single-spanning proteins
**NAKH920105**	AA composition of MEM of single-spanning proteins
**NAKH920108**	AA composition of MEM of multi-spanning proteins
**OOBM770101**	Average non-bonded energy per atom
**OOBM850101**	Optimized beta-structure-coil equilibrium constant
**OOBM850102**	Optimized propensity to form reverse turn
**OOBM850105**	Optimized side chain interaction parameter
**PRAM900101**	Hydrophobicity
**RACS770102**	Average reduced distance for side chain
**RADA880108**	Mean polarity
**TANS770101**	Normalized frequency of alpha-helix
**TANS770103**	Normalized frequency of extended structure
**TANS770108**	Normalized frequency of zeta R
**TANS770109**	Normalized frequency of coil
**VELV850101**	Electron-ion interaction potential
**ZIMJ680101**	Hydrophobicity
**ZIMJ680103**	Polarity
**ZIMJ680104**	Isoelectric point
**WOLR790101**	Hydrophobicity index
**ENGD860101**	Hydrophobicity index
**FASG890101**	Hydrophobicity index
