# Supplementary material for: Predicting human protein subcellular localization by heterogeneous and comprehensive approaches
Source: PLoS One. 2017 Jun 28;12(6):e0178832. doi: 10.1371/journal.pone.0178832 (PMC5489166; doi:10.1371/journal.pone.0178832)
Supplement: S2 Table — The number of SVM models and the ratio of negative and positive data in the first layer. (PDF) [file pone.0178832.s011.pdf]

Supplementary table 2 The number of SVM models and the ratio of negative and positive data in the first layer

Subcellular location	Number of Models	Best P/N ratio*
Cell membrane	3	1 : 1.1
Cytoplasm	5	1 : 1.0
ER/Golgi	7	1 : 1.5
Mitochondrion	9	1 : 1.4
Nucleus	3	1 : 1.0
Extracellular	5	1 : 1.5

* P/N ratio: the ratio of positive data to negative data
