# Supplementary material for: Predicting human protein subcellular localization by heterogeneous and comprehensive approaches
Source: PLoS One. 2017 Jun 28;12(6):e0178832. doi: 10.1371/journal.pone.0178832 (PMC5489166; doi:10.1371/journal.pone.0178832)
Supplement: S1 Algorithm — The procedure of regular-mRMR in REALoc. (PDF) [file pone.0178832.s012.pdf]

Supplementary algorithms 1

GO Regular-mRMR function- minimum Redundancy Maximum Relevance Feature Selection (mRMR) is a method for choosing features and simultaneously preventing features with similar characteristics appearing more than once. The features are placed in order of importance. However, this method produces different results depending on different order of original data. Therefore, Regular-mRMR can be used to solve this problem. The algorithm was as follows:

```
GO regular-mRMR fuction{
    for i＝1 to 5 times{
        re-sort GO_numbers;
        run mRMR;
        for j＝1 to 50 ranks{
            score_GO_numbers＝score_GO_numbers＋（50－j＋1）;
        }
    }
    score_GO_numbers＝score_GO_numbers／5 times;
    sort GO_numbers by score_GO_numbers;
    return top 50 GO_numbers;
}
```
